# NH_2_-Modified UiO-66: Structural Characteristics and Functional Properties

**DOI:** 10.3390/molecules28093916

**Published:** 2023-05-05

**Authors:** Konstantin L. Timofeev, Sergei A. Kulinich, Tamara S. Kharlamova

**Affiliations:** 1 Laboratory of Catalytic Research, Tomsk State University, 634050 Tomsk, Russia; kvintkl@gmail.com; 2 Research Institute of Science & Technology, Tokai University, Hiratsuka 259-1292, Kanagawa, Japan

**Keywords:** UiO-66, UiO-66-NH_2_, structural peculiarities, basic properties, IEP

## Abstract

The development of new functional materials based on metal–organic frameworks (MOFs) for adsorption and catalytic applications is one of the promising trends of modern materials science. The Zr-based MOFs, specifically UiO-66, are considered as the supports for metallic catalysts for the 5-hydroxymethylfurfural platform molecule reduction into valuable products. The present work focused on the effect of NH_2_ modification of UiO-66 on its structure and functional properties. The samples were prepared by a solvothermal method. The structure of the obtained materials was studied by X-ray diffraction, IR spectroscopy, UV–visible spectroscopy, and low-temperature nitrogen adsorption. Basic properties were investigated by HCl and CH_3_COOH adsorption, and electrokinetic properties were studied by electrophoretic light scattering. UiO-66-NH_2_ samples with different contents of aminoterephthalate linkers were successfully prepared. A gradual decrease in the specific surface area and the fraction of micropores with a diameter of ~0.9 nm was observed with an increase in the aminoterephthalate content. A proportional increase in the total number of basic sites in UiO-66-NH_2_ samples was established with an increase in the aminoterephthalate content up to 75%. At the same time, a noticeable decrease in the total number of basic sites and an increase in their strength with higher aminoterephthalate content was observed.

## 1. Introduction

The use of biomass as an easily renewable source is becoming an increasingly attractive and promising way to obtain a wide range of valuable compounds [1,2]. Among the various biomass-derived products, 5-hydroxymethylfurfural (5-HMF) is considered one of the most promising platform molecules [3,4,5]. Supported noble metals (Au, Pd, Pt, Ag, etc.) and bimetallic compositions on the basis thereof are the most abundant and well-proven catalytic materials proposed for HMF reduction [4,5,6]. However, despite the progress made in the development of catalysts for HMF transformation, further catalyst improvement is required, and a support can notably affect the performance.

Recently, metal–organic frameworks (MOFs) have attracted increasing attention in various fields due to their high porosity and variety of structures [7]. Zirconium terephthalate UiO-66 deserves special interest due to its unprecedented stability [8,9]. Modification of UiO-66 by introducing various functional groups and/or metal centers into its structure creates additional functionality of the resulting new materials. The modification of MOFs with NH_2_ groups makes it possible to change its acid–base and optical properties as well as to create additional functionality for stabilization of metal particles or further modification [10,11,12,13,14]. Specifically, the Pd/MIL-101(Al)-NH_2_ catalyst for the selective 5-HMF hydrogenation to 2,5-dihydroxymethyl-tetrahydrofuran (DHMTHF) was prepared using a direct anionic exchange approach and subsequent soft reduction [10], with the presence of free amine moieties being suggested to play the key roles in the formation of uniform and well-dispersed palladium nanoparticles on the support. Moreover, the observed high selectivity towards DHMTHF was closely related to the cooperation between the metallic site and the free amine moiety on the MOF support in the Pd/MIL-101(Al)-NH_2_ catalyst.

A similar approach was used to prepare the Pd@NH_2_-UiO-66 catalyst for the vanillin hydrodeoxygenation in water [11]. A photoinduction method including Cu species adsorption by NH_2_ groups of the UiO-66-NH_2_ support followed by their anchoring after irradiation with visible light was successfully used to construct single-atom Cu/UiO-66-NH_2_ as a robust photocatalyst for CO_2_ conversion to liquid fuels [15]. In this case, Cu species anchored were optimized the structure of photocatalysts at the atomic level to facilitate the separation of electron−hole pairs. The Cu(II)@UiO-66-NH_2_ and Cu(0)@UiO-66-NH_2_ catalytic materials were generated via the simple impregnation of nano-sized UiO-66-NH_2_ with Cu(OAc)_2_ solution and the subsequent metal ion reduction process, respectively [16]. Spherical-shaped copper particles uniformly distributed in Cu(0)@UiO-66-NH_2_ material with an average size of 4–6 nm were revealed by HRTEM, indicating the formation of Cu NPs primarily on the external surface than in the pores of the MOFs characterized by the structure with cavities of ~9 and ~11 Å. Highly dispersed Au nanoparticles were immobilized on amino-functionalized UiO-66-NH_2_ MOFs via the absorption/reduction method in solution [17], with the amino functionality of the MOF being considered to rapidly coordinate with HAuCl_4_ and act as the Au(0) precursor. However, the primary formation of Au NPs coated on the external surface of the MOFs was reported for Au@UiO-66-NH_2_ composites prepared using similar approaches [18,19].

The formation of bimetallic composites based on UiO-66-NH_2_ was also described. Thus, Au−Pd@UiO-66-NH_2_ heterogeneous catalysts for reductive amination of benzaldehyde with extremely low Pd loading and Au/Pd@UiO-66-NH_2_ (Au:Pd = 1:1, 1:2, 2:1) photocatalysts for the liquid-phase Suzuki–Miyaura coupling reaction were prepared by a one-pot absorption–reduction method [20,21]. However, the confirmation of the formation of alloyed particles in both cases was rather speculative. The formation of Au particles was revealed by XRD and UV–vis spectroscopy data for Au/Pd@UiO-66-NH_2_ photocatalysts [21].

Therefore, the results reported for metal-loaded metal–organic frameworks, specifically metal@NH_2_-UiO-66 materials, strongly differ even for similar preparation approaches, with the size, composition, and location of metal particles strongly affecting the material performance [22,23]. This indicates that such strategies are still developed by trial and error to some extent, and an in-depth understanding of the mechanism of formation is still lacking, especially for bimetallic systems. Specifically, the Zr_6_O_4_(OH)_4_ clusters can also play the key role in the metal particle formation in UiO-66-NH_2_-based materials along with the amino group. Further, to create the bifunctional systems with redox and acid–base species, it is important to study acid–base properties of the materials under conditions close to those of the reaction medium. Thus, more extensive studies of the structure peculiarities and functional properties of the NH_2_-modified UiO-66 MOFs are highly desired for purposeful design of the UiO-66-based bimetallic catalysts.

The present work is focused on the study of crystal and pore structure peculiarities and functional properties of NH_2_-modified UiO-66 considered as a support for PdCu and PdAu catalysts for 5-HMF reduction in water solutions.

## 2. Results and Discussion

UiO-66 and UiO-66-NH_2_-X (where X is a fraction of aminoterephthalate linkers) samples with different contents of aminoterephthalate linkers were prepared by a solvothermal method. The morphologies of UiO-66 and UiO-66-NH_2_-X crystals were characterized by scanning electron microscopy (SEM). The structure of the obtained materials was studied by X-ray diffraction (XRD), IR spectroscopy, UV–vis spectroscopy, and low-temperature N_2_ adsorption. Basic properties were investigated by HCl and CH_3_COOH adsorption from water solutions, and electrokinetic properties were studied by electrophoretic light scattering.

### 2.1. Structural Characteristics

#### 2.1.1. SEM and X-ray Diffraction

**Figure 1:**
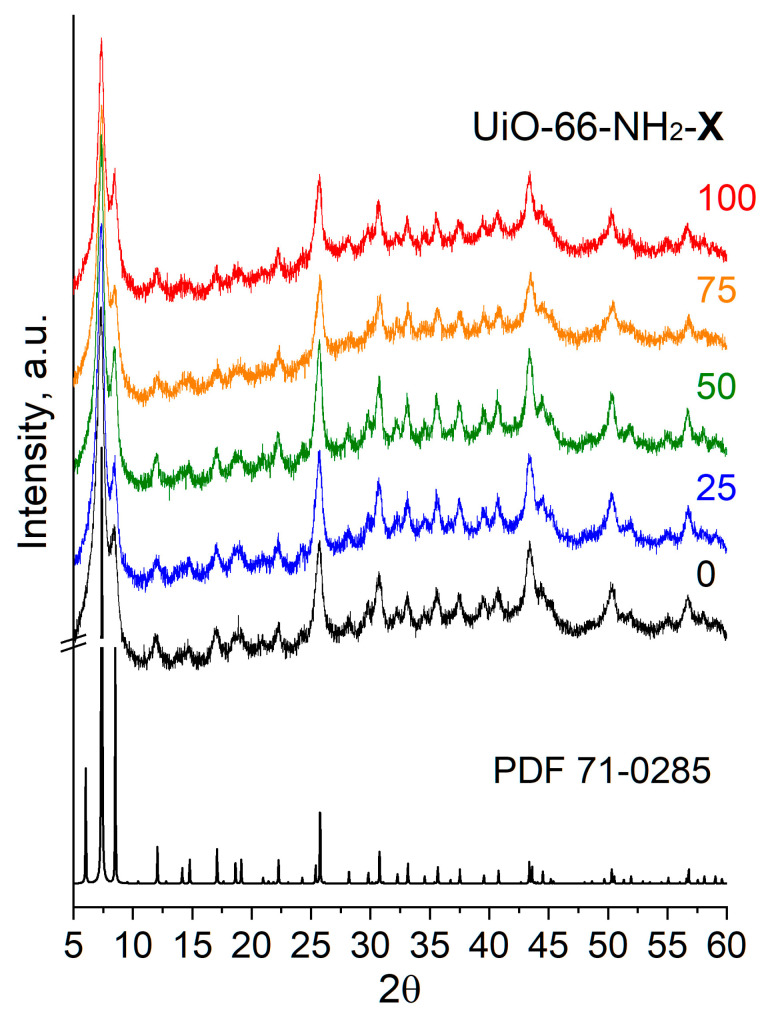
XRD patterns for UiO-66 (X = 0) and UiO-66-NH_2_-X samples.

The samples prepared were formed by small (<300 nm) crystals (Figure S1). The UiO-66 crystal structure was confirmed by XRD for all samples obtained. The XRD patterns of the samples (Figure 1) contain characteristic reflections of the cubic UiO-66 (ICDD PDF-4 card #71-0285), and Table 1 represents a lattice parameter, *a*, and a unit cell volume, *V*. According to the results obtained, the increase in the aminoterephthalate content in the UiO-66 structure is accompanied by some lattice compression. Specifically, the unit cell volume gradually decreases from 9077 Å^3^ for the UiO-66 sample to 8960 Å^3^ for the UiO-66-NH_2_-75 sample as the aminoterephthalate content is increased. A slight increase in the unit cell in volume is observed for the UiO-66-NH_2_-100 sample as compared with the UiO-66-NH_2_-75 sample but it remains lower than that for other samples. These results are consistent with those presented in [24,25,26], where the XRD reflection shift to higher values is observed for UiO-66-NH_2_ MOFs.

#### 2.1.2. IR Spectroscopy

**Figure 2:**
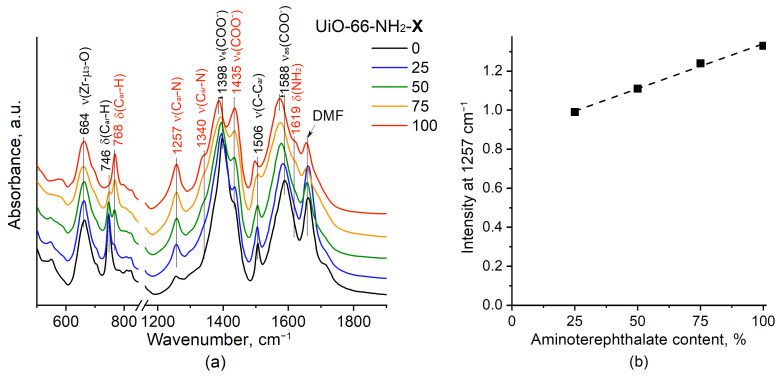
IF spectra for UiO-66 (X = 0) and UiO-66-NH_2_-X samples (**a**) and dependence of ν(C_ar_–N) band intensity on the aminoterephthalate content in the sample (**b**). DMF is dimethylformamide.

To confirm the formation of NH_2_-modified UiO-66 and study its structural peculiarities, the samples were studied by IR spectroscopy. Figure 2 shows the IR spectra for the samples containing a number of characteristic bands within the range from 500 to 1900 cm^−1^. Figure S2 shows the expanded views of subregion 2600–3200 cm^−1^ (Supplementary Materials).

The spectrum for the pristine UiO-66 contains the bands at 1397 and 1589 cm^−1^ assigned to COO^−^ stretching vibrations, at 1506 and 746 cm^−1^ associated with C–C and C–H vibrations in the benzene ring, respectively, and at 664 cm^−1^ attributed to μ_3_–O stretching in Zr_6_O_4_(OH)_4_ clusters [27,28]. A band at 1660 cm^−1^ is due to the dimethylformamide (DMF) residual [29].

For the UiO-66-NH_2_ samples, new bands at 1257 and 1340 cm^−1^ assigned to C–N stretching vibrations as well as at 1619, 3367, and 3453 cm^−1^ assigned to NH_2_ group vibrations appear (see subregion 2600–3200 cm^−1^ in Figure S2) [27]. Further, the appearance of a new band at 768 cm^−1^ assigned to C–H vibrations in the NH_2_-substituted benzene ring as well as a gradual shift in COO^−^ stretching vibration bands to lower wavenumbers accompanied by a splitting of symmetric COO^−^ stretching vibrations (decrease in the intensity of the band at 1387–1398 cm^−1^ and appearance of a new band at 1435 cm^−1^) is observed due to the NH_2_ group effect. The intensity of the nonoverlapping band at 1257 cm^−1^ proportionally increases with the increase in the aminoterephthalate content in the sample, indicating the formation of UiO-66-NH_2_ MOFs with different amounts of NH_2_ groups (Figure 2b).

#### 2.1.3. UV–Vis Spectroscopy

**Figure 3:**
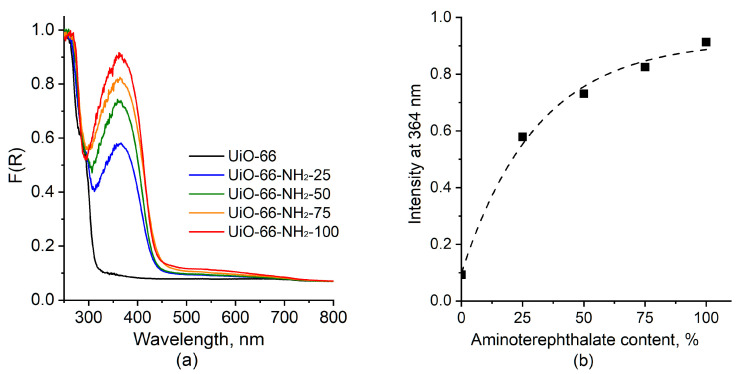
UV-vis spectra for UiO-66 (X = 0) and UiO-66-NH_2_-X samples (**a**) and dependence of the intensity at 364 nm on the aminoterephthalate content in the sample (**b**).

The optical properties of the samples were studied by UV–vis spectroscopy. Figure 3 shows UV–vis spectra for UiO-66 and UiO-66-NH_2_-X samples. The spectrum for the former is characterized by the absorption below 320 nm caused by π-π* electronic transition of the biphenyl linker system [24,30]. In spectra for the UiO-66-NH_2_-X sample, an absorption band with a maximum at 364 nm caused by the electronic transition from the nonbonding to antibonding molecular orbital of aminoterephthalate (n → π*) is additionally present [24,30,31]. The intensity of this band increases with an increase in the aminoterephthalate content according to the Beer–Lambert law (Figure 3b), which is consistent with the IR spectroscopy data, indicating the formation of UiO-66-NH_2_ MOFs with different numbers of NH_2_ groups.

#### 2.1.4. Textural Characteristics

**Figure 4:**
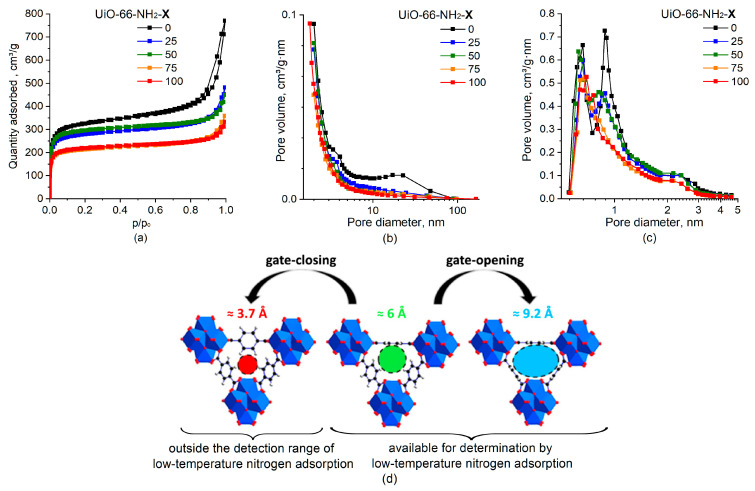
Adsorption isotherms (**a**) and mesopore size distribution according to the BJH desorption method (**b**) and micropore size distribution according to the Horvath–Kawazoe method (**c**) for UiO-66 (X = 0) and UiO-66-NH_2_-X samples. Change in pore entrance size as a result of the three oriented benzene rings flipping around the C_2_ symmetry axis (**d**). Adapted with permission from [36]; copyright 2017 American Chemical Society.

Figure 4 shows the isotherms of low-temperature N_2_ adsorption–desorption for UiO-66 and UiO-66-NH_2_-X samples, and Table 1 shows the values of specific surface area (SSA) and pore volumes of micropores (V_HK_) and mesopores (V_BJH_).

According to the IUPAC classification [32], all samples are characterized by type I isotherms typical for microporous materials with a hysteresis loop of the H4 type (Figure 4a). The steep uptake at rather low relative pressures (<0.05) is due to the micropore filling caused by the enhanced adsorbent–adsorptive interactions in narrow micropores. The presence of the hysteresis loop in the range of 0.8–1.0 p/p_o_ indicates that the sample contains mesopores due to the defects in the UiO-66 structure and gaps between the particles in aggregates. In general, a gradual decrease in the specific surface area and pore volume of the samples is observed with an increase in the aminoterephthalate content in the sample (Table 1). The deviations from the monotonic decrease in the SSA and pore volume do not exceed a 10% error when they are determined from the low-temperature nitrogen adsorption data. The observed decrease in SSA and pore volume are attributed to both reduced available free space and increased overall weight of the UiO-66-NH_2_-X samples.

The unmodified UiO-66 sample is characterized by a bimodal distribution of micropores with maxima at ~0.9 nm and ~0.6 nm (Figure 4c) that is typical for the UiO-66 [33,34,35]. The observed bimodal distribution is consistent with a change in the size of the pore entrance formed by the terephthalate bridges between the Zr_6_O_4_(OH)_4_ sites as a result of a change in the orientation of benzene rings due to the flips around the C_2_ symmetry axis [36]. Specifically, the benzene rings were shown to flip, changing the pore entrance size to 3.7, 6, and 9.2 Å (Figure 4d), with latter two sizes being available for determination by the low-temperature N_2_ adsorption and the smaller pores being outside the detection range of the method.

For the UiO-66-NH_2_-X samples, a gradual decrease in the volume for pores with a diameter of ~0.9 nm is observed in the micropore size distribution as the aminoterephthalate content increases (Figure 4c). For the UiO-66-NH_2_-100 sample, only micropores with a diameter of ~0.6 nm is present. The observed change in the pore size distribution was associated with the hindered flips of the NH_2_-substututed benzene rings around the C_2_ symmetry axis and the predominant orientation of benzene rings corresponding to micropore sizes of ~0.6 nm in the detection range of the low-temperature N_2_ adsorption at least at −195.8 °C. This does not exclude orientation of benzene rings corresponding to micropore sizes with the smaller entrance radius (outside the detection range) as well as the possibility of rotation of the NH_2_-substututed benzene ring at higher temperatures. The effect of NH_2_ groups, which are larger than H atoms, on micropore size distribution for UiO-66-NH_2_-X samples also cannot be ruled out [37].

### 2.2. Functional Proreties

#### 2.2.1. Electrokinetic Properties

The water dispersions of UiO-66 and UiO-66-NH_2_-X samples were characterized by an initial pH of ~3.7 and a zeta potential of 30.7 ± 1.2 mV independent of their composition. The pH increase resulted in rapid decrease in the surface charge (Figure S3, exemplified by the UiO-66 and UiO-66-NH_2_-75 samples), with the IEP being at a pH of 4.4–4.7. Similar results were reported for UiO-66-NH_2_ [38]. Comparable zeta potential dependences for UiO-66 and UiO-66-NH_2_ MOFs (but characterized by the IEP at a pH of 5.6) were observed in [39]. The observed difference in the IEP can be caused by differences in experimental conditions, including temperature, the error in pH measurements, and the presence of impurities [40].

Independence of electrokinetic properties of the samples on their composition indicates that those are primarily related to Zr_6_O_4_(OH)_4_ clusters due to the formation of surface -ZrOH_2_^+^, -ZrOH and -ZrO^−^ groups in water at different pH but not to linkers.

#### 2.2.2. Basic Properties

**Figure 5:**
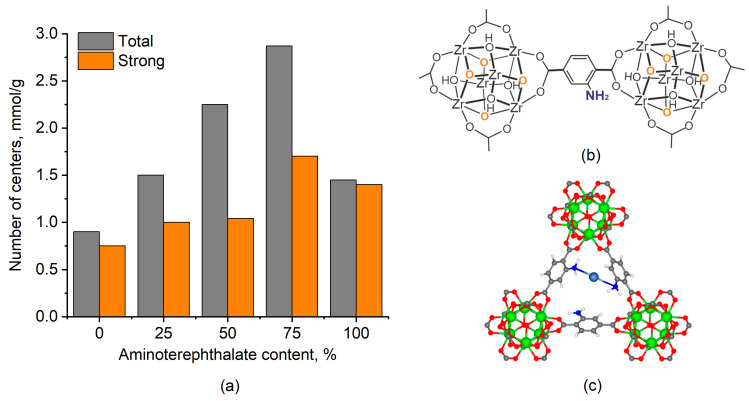
Dependence of the number of basic sites on the aminoterephthalate content in UiO-66 (X = 0) and UiO-66-NH_2_-X samples (**a**). Illustrative depiction of basic sites in UiO-66-NH_2_-X samples: basic sites in Zr_6_O_4_(OH)_4_ clusters indicated by orange and NH_2_ group of aminoterephthalate linker indicated by blue (**b**). The local atomic structure model of multicoordinated atoms exemplified by isolated Cu species coordinated to two N atoms on UiO-66-NH_2_ according to [15] (**c**). Adapted with permission from [15]; copyright 2020 American Chemical Society.

The basic properties of the UiO-66 and UiO-66-NH_2_-X samples were studied by the adsorption of acids from aqueous solutions. HCl and CH_3_COOH were used as strong and weak acid to determine the total number of basic sites and strong basic sites, respectively. Figure 5a shows the results obtained. The pristine UiO-66 is characterized by the relatively low (0.9 mmol/g) number of basic sites predominantly characterized by strong basicity. These basic sites are assigned to the basicity of Zr_6_O_4_(OH)_4_ clusters (Figure 5b) [41,42,43].

A gradual increase in the total number of basic sites up to 2.75 mmol/g with the increase in the aminoterephthalate content in the UiO-66-NH_2_-X samples up to 75% is observed but the complete replacement of terephthalate in the UiO-66-NH_2_-100 sample with aminoterephthalate is accompanied by a sharp decrease in the total number of basic sites up to 1.5 mmol/g. The number of strong basic sites increases insignificantly up to the aminoterephthalate content in UiO-66-NH_2_-X sample of 50%, which is consistent with low basicity of aryl amins due to the nitrogen electron pair delocalization [44]. However, at the higher aminoterephthalate content, the number of strong basic sites notably increases, and almost all sites in the UiO-66-NH_2_-100 sample are strong. The observed decrease in the total number of basic sites accompanied by the increase in the strong site fraction was associated with the formation of sites related to a proton multicoordinated between several nitrogen atoms [45]. The formation of multicoordinated species in MOFs was recently revealed for isolated Cu species anchored on UiO-66-NH_2_ by element-selective X-ray absorption fine structure measurements [15]. Specifically, the results obtained illustrated that the isolated Cu atoms were coordinated to two N atoms with atom distances of 1.97 Å (Cu−N). The local atomic structure model of such Cu species is shown in Figure 5c. In case of the proton, the sharp decrease in the total number of basic sites indicates that it requires to be coordinated to more N atoms to form multicoordinated species.

### 2.3. UiO-66-NH_2_ as a Support

The UiO-66-NH_2_ MOFs attracts attention as a support for the fabrication of metal@MOF hybrid catalysts because of its high thermal and chemical stability, large accessible pore volume, and the amine groups within UiO-66-NH_2_ serving as the coordination sites for metal ions. The IR and UV–vis spectroscopy data indicate that the amount of NH_2_ moities can be easily controlled by varying the ratio of terephthalic and aminoterephthalic acids during MOF synthesis by the solvothermal method. This provides additional opportunities to control the distribution and size of metal particles formed with the participation of NH_2_ groups.

Various mechanisms for the metal precursor anchoring with the participation of NH_2_ groups are described in the literature, including electrostatic interaction between the -NH_3_^+^ cation formed via amino group protonation and the anion (-NH_3_^+^{PdCl_4_]^2−^) [10,21], the coordination interaction between the NH_2_ group and the metal cation by a lone pair of electrons on nitrogen [46], etc. At the same time, according to the studies of basic properties of UiO-66-NH_2_-X, the amino groups in the UiO-66-NH_2_-X exhibit weak basic or electron-donating properties, which limits the adsorption of precursors with their participation. The electron-accepting ability of the metal ions, and, as a result, their adsorption, can be controlled by pH, temperature, and concentrations of initial ions [46]. Further, the fraction of amino groups in UiO-66-NH_2_-X MOFs can also affect the sorption capacity and adsorption strength due to multicenter adsorption as it was revealed for proton adsorption in this study and for Cu single atoms in UiO-66-NH_2_ [15]. This understanding is consistent with the formation of Cu NPs for Cu(0)@UiO-66-NH_2_ with a Cu content of 3.5 wt% [16] and Cu single atoms for Cu SAs@UiO-66-NH_2_ with a Cu content of 0.3 wt% [15]. In the case of bimetallic systems, it is also necessary to consider the possible competitive sorption of precursor ions.

The results of the electrokinetic studies indicate that Zr_6_O_4_(OH)_4_ clusters in the UiO-66-NH_2_-X materials can also play an important role in the interaction with the precursors of deposited metals. Specifically, at low pH, the UiO-66-NH_2_-X samples are characterized by a high positive charge due to the formation of surface -ZrOH_2_^+^ species, which should promote the adsorption of AuCl_4_^−^ and PdCl_4_^2−^ anions.

Therefore, the results obtained for the UiO-66-NH_2_-X provide insight for the purposeful design of metal@MOF hybrid catalysts.

## 3. Materials and Methods

### 3.1. Materials Preparation

UiO-66 and UiO-66-NH_2_ samples (UiO-66-NH_2_-X, where X is a fraction of aminoterephthalate linkers) were prepared by a solvothermal method using ZrO(NO_3_)_2_ × H_2_O as a precursor of Zr and terephthalic acid and/or 2-aminoterephthalic acid as linkers, HCl as a modulator, and DMF as a solvent. ZrO(NO_3_)_2_ × H_2_O was dissolved in DMF with HCl at 120 °C. Terephthalic acid and/or 2-aminoterephthalic acid was dissolved in DMF at 120 °C in a round-bottomed flask. Then, the ZrO(NO_3_)_2_ solution was added to the acid solution and heated at 120 °C and vigorous stirring for 1 h in a closed flask for hydrothermal reaction. After cooling, the resulting solid was filtered, immersed twice in DMF for 12 h followed by filtration to remove unreacted terephthalic acid. Then, it was immersed in ethanol for 12 h, and in acetone for 12 h to remove DMF, and dried at room temperature.

### 3.2. Low-Temperature Nitrogen Adsorption

Low-temperature nitrogen adsorption at −196 °C was carried out using the 3Flex (Micromeritics, Norcross, GA, USA) gas-adsorption analyzer of specific surface area and porosity. Prior to the experiments, the samples were degassed at 200 °C under vacuum (10^−2^ Torr) for 2 h using the VacPrep Degasser (Micromeritics, Norcross, GA, USA). The specific surface area was determined by the Brunauer–Emmett–Teller (BET) method. The mesopore size distribution was determined by the Barrett–Joyner–Halenda (BJH) desorption method, and the micropores size distribution was determined by the Hovarth–Kowazoe method.

### 3.3. X-ray Diffraction

XRD patterns of the samples were obtained on the X-ray diffractometer XRD-7000 (Shimadzu, Japan) with a monochromatic CuKα radiation (1.54 Å) in the 2θ range of 5–60° and a scanning rate of 0.02°/s. The data were obtained using the Bragg–Brentano geometry. Crystalline Si (*a* = 5.4309 Å, λ = 1.540562 Å) was used as an external standard for diffractometer calibration. The phase composition of the samples was analyzed using the PDF-4 database (Release 2021 RDB).

### 3.4. IR Spetroscopy

IR spectra were obtained on the Tensor 27 IR-Fourier spectrometer (Bruker, Germany) in the transmission mode using the KBr pressing method in the range of 350–4000 cm^−1^ with a resolution of 4 cm^−1^.

### 3.5. UV–Vis Spectroscopy

Absorption spectra in the UV–visible region were recorded on the Cary100 spectrophotometer (Varian, Mulgrave, VIC, Australia) in a diffuse reflection (DR) mode using the DRA-CA-30I accessory (Labsphere, USA) in the wavelength range of 300–800 nm with a step of 1 nm using the quartz cuvette with an optical path length of 2 mm. MgO was used as a reference sample. The samples were mixed with MgO prior to study in a mass ratio sample/MgO = 1/9. The reflection spectra were transformed into the absorption spectra using the Kubelka–Munk function:

F(R) = (1 − R)^2^/(2R),

where R is the reflection factor.

### 3.6. Electrophoretic Light Scattering

The electrokinetic properties of the dispersions were studied by electrophoretic light scattering (ELS) using the phase analysis light scattering (PALS) technique on the Omni S/N analyzer (Brookhaven, NY, USA) equipped with the BI-ZTU autotitrator (Brookhaven, NY, USA). For study, the samples were dispersed in a distilled water at a concentration of 25 mg/L using ultrasound for 3 min. To determine the pH of the isoelectric point (IEP), the dispersion was titrated using the diluted (0.001 and 0.1 mol/L) KOH solutions.

### 3.7. Acid Adsorption

The basic properties of the samples were studied by the adsorption of acids from aqueous solutions [47,48]. HCl was used to determine the total number of basic sites, while CH_3_COOH was used to determine the strong basic sites. For the study, 0.5 g of the sample was suspended in 50 mL of an aqueous solution of HCl or CH_3_COOH (0.1 mol/L). The suspension was thoroughly stirred for 1 h at room temperature. Then, the suspension was filtered, and the filtrate was titrated potentiometrically with an aqueous KOH solution (0.1 mol/L). The titration potential was measured with the pH meter/ionometer ITAN (LLC SPE Tomanalit, Tomsk, Russia). The basicity was calculated as follows:

$$N=\frac{(C_{0}-C_{1})V_{0}}{m}$$

where *N* is the concentration of basic sites, mol/g; *C*_0_ is the concentration of the acid solution before adsorption, mol/L; *V*_0_ is a volume of the acid solution used, L; *C*_1_ is the concentration of the acid solution after adsorption, mol/L; *m* is a weight of the sample.

### 3.8. Scanning Electron Microscopy

The morphology of the materials was studied using a Vega 3 SBH scanning electron microscope (SEM) (Tescan, Brno, Czech Republic).

## 4. Conclusions

A series of NH_2_-modified UiO-66 samples with different contents of aminoterephthalate linkers was studied. The amount of NH_2_ moieties in UiO-66-NH_2_ can be easily controlled by varying the ratio of terephthalic and aminoterephthalic acids during MOF synthesis. The UiO-66 crystal structure was confirmed by XRD for all materials obtained, with the increasing fraction of aminoterephthalate in the UiO-66-NH_2_ framework being confirmed by the IR and UV–vis spectroscopies. The UiO-66 modification with NH_2_ groups accompanied by changes in pore structure, optical and basic/electron-donating properties of the materials can affect the characteristics of the catalyst on the basis thereof.

## Figures and Tables

**Table 1:** XRD (lattice parameter *a*; unit cell volume *V*) and low-temperature adsorption data (specific surface area (SSA); specific volume of micropore (V_HK_) and mesopore (V_BJH_)).

Sample	SG	*a*, Å	*V*, Å^3^	SSA, m^2^/g	V_HK_, cm^3^/g	V_BJH_, cm^3^/g
UiO-66	Pm-3m	20.86	9077	1269	0.50	0.83
UiO-66-NH_2_-25	Pm-3m	20.83	9037	1091	0.43	0.40
UiO-66-NH_2_-50	Pm-3m	20.83	9037	1158	0.45	0.31
UiO-66-NH_2_-75	Pm-3m	20.77	8960	825	0.33	0.28
UiO-66-NH_2_-100	Pm-3m	20.80	8999	854	0.34	0.24

## Data Availability

Not applicable.

## Supplementary Materials

The following supporting information can be downloaded at: https://www.mdpi.com/article/10.3390/molecules28093916/s1, Figure S1: Typical SEM images of UiO-66 and UiO-66-NH2-75 samples. Figure S2: IR spectra for UiO-66 (X = 0) and UiO-66-NH_2_-X samples in subregion 2600–3200 cm^−1^. Figure S3: Zeta potential dependences for UiO-66 and UiO-66-NH_2_-75 samples on pH.
