# Supplementary material for: NH2-Modified UiO-66: Structural Characteristics and Functional Properties
Source: Molecules. 2023 May 5;28(9):3916. doi: 10.3390/molecules28093916 (PMC10180438; doi:10.3390/molecules28093916)
Supplement: Supplementary file 1 [file molecules-28-03916-s001.zip › molecules-2369927-supplementary.pdf]

molecules

MDPI

Supplementary Materials

NH_2-Modified UiO-66: Structure, Textural Characteristics, and Functional Properties

Konstantin L. Timofeev [1], Sergei A. Kulinich [2], Tamara S. Kharlamova [1,*]

[1] Tomsk State University, Lenin Ave. 36, 634050 Tomsk, Russia
[2] Research Institute of Science & Technology, Tokai University, Hiratsuka 259-1292, Kanagawa, Japan
* Correspondence: skulinich@tokai-u.jp (S.A.K.); kharlamova83@gmail.com (T.S.K)

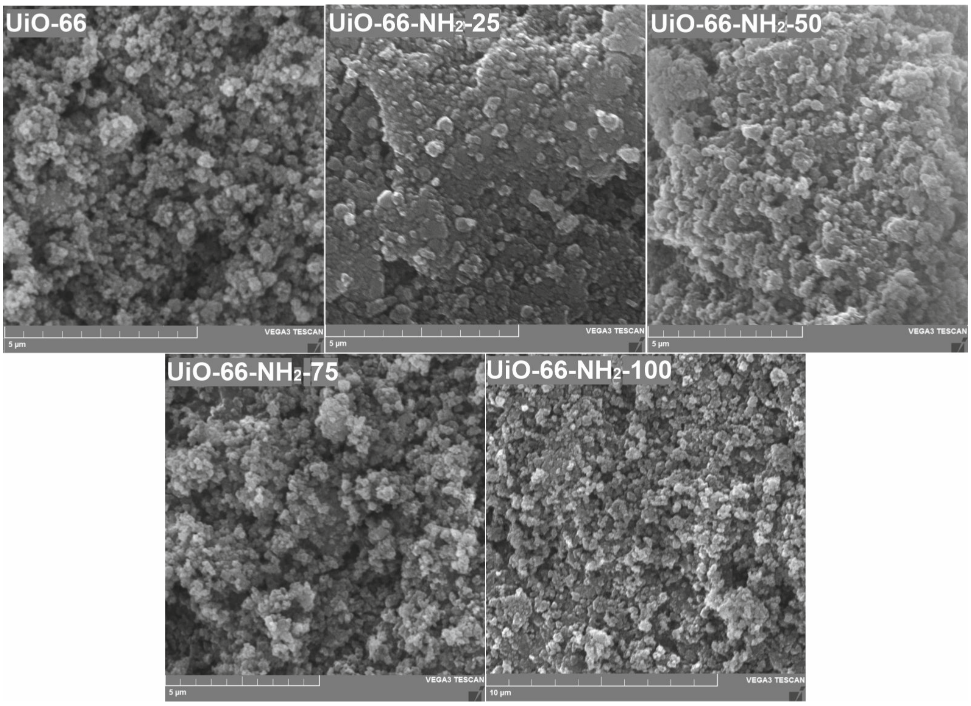

Figure S1. Typical SEM images of UiO-66 and UiO-66-NH_2-75 samples.

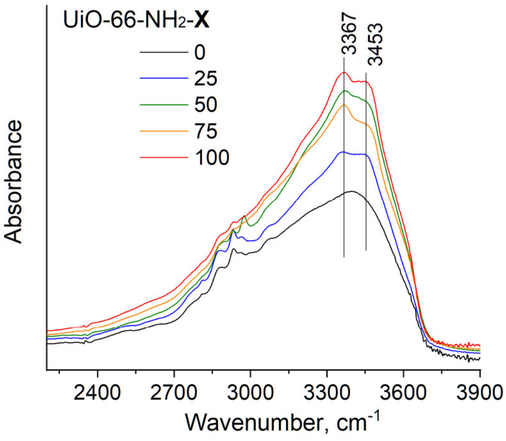

Figure S2. IR spectra for UiO-66 (X=0) and UiO-66-NH_2-X samples in sub-region 2600–3200 cm^{-1}.

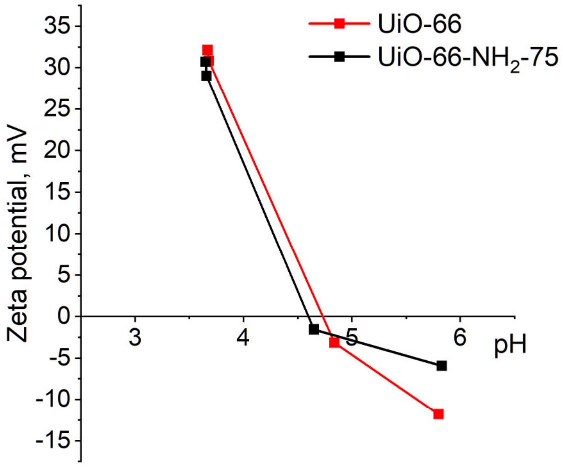

Figure S3. Zeta potential dependences for UiO-66 and UiO-66-NH_2-75 samples on pH.
